# Modeling Recovery Housing Retention and Program Outcomes by Justice Involvement among Residents in Virginia, USA: An Observational Study

**DOI:** 10.1177/0306624X241254691

**Published:** 2024-06-10

**Authors:** Arun Sondhi, Adela Bunaciu, David Best, Emily A. Hennessy, Jessica Best, Alessandro Leidi, Anthony Grimes, Matthew Conner, Robert DeTriquet, William White

**Affiliations:** 1 Therapeutic Solutions (Addictions) Ltd., London, UK; 2 Department of Psychology, School of Humanities, Social Science and Law, University of Dundee, Dundee, UK; 3 Centre for Addiction Recovery Research, Leeds Trinity University, Leeds, UK; 4 Massachusetts General Hospital and Harvard Medical School, Boston, MA, USA; 5 Recovery Outcomes Institute, Boynton Beach, Florida, USA; 6 Statistical Services Centre, Reading, UK; 7 Virginia Association of Recovery Residences, Richmond, VA, USA; 8 Chestnut Health Systems, Normal, Illinois, USA

**Keywords:** recovery, recovery residences, time to event analysis, retention, outcomes

## Abstract

Living in recovery housing can improve addiction recovery and desistance outcomes. This study examined whether retention in recovery housing and types of discharge outcomes (completed, “neutral,” and “negative” outcomes) differed for clients with recent criminal legal system (CLS) involvement. Using data from 101 recovery residences certified by the Virginia Association of Recovery Residences based on 1,978 individuals completing the REC-CAP assessment, competing risk analyses (cumulative incidence function, restricted mean survival time, and restricted mean time lost) followed by the marginalization of effects were implemented to examine program outcomes at final discharge. Residents with recent CLS involvement were more likely to be discharged for positive reasons (successful completion of their goals) and premature/negative reasons (e.g., disciplinary releases) than for neutral reasons. Findings indicate that retention for 6–18 months is essential to establish and maintain positive discharge outcomes, and interventions should be developed to enhance retention in recovery residents with recent justice involvement.

## Introduction

Substance addiction has pervasive and devastating personal, social, and economic consequences. Fortunately, achieving sustained recovery is not uncommon, as over half of those suffering from chronic addiction achieve sustained recovery (Best & Lubman, 2012). Recovery from addiction is demanding on its own. However, those attempting to desist from offending face greater challenges as the criminal legal system (CLS) often creates barriers to, for instance, engaging in employment, education, safe and stable housing, and reintegration into society (Polcin, 2018). Studies have shown that reoffending is common after release from prison (Yukhnenko et al., 2019). However, the reoffending rate is even greater among those suffering from addiction (Holloway et al., 2022; Zgoba et al., 2020). Since finding safe and stable housing is a critical challenge in desistance, recovery houses can simultaneously support both recovery and desistance by providing alcohol- and drug-free housing and supporting reintegration into the community (Polcin, 2018).

### Overlap Between Offending and Substance Misuse, and Recovery and Desistance

Evidence suggests a considerable overlap between CLS involvement and substance use. For example, a study in England and Wales found that 69% of individuals arrested had used at least one illegal drug (Bennett & Holloway, 2004), while a US study found that just over half of those incarcerated had used drugs during the month before offending (Maruschak et al., 2021). Offending and substance use are highly stigmatized and associated with societal responses that hinder a successful everyday life (Best et al., 2016; Van Roeyen et al., 2017). For instance, barriers include a lower likelihood of acquiring safe and stable housing and accessing mortgages, fewer employment prospects and earning potential, and more difficulty accessing insurance resulting in greater unmet needs and reduced personal, social, and community capital (Henley, 2014; Neale et al., 2016).

While healing from addiction and desisting from offending behaviors are distinct processes of change, there are multiple areas where recovery and desistance overlap (Van Roeyen et al., 2017). SAMHSA (2012, p. 3) defined recovery as “*a process of change through which individuals improve their health and wellness, live a self-directed life, and strive to reach their full potential.”* In turn, desistance has been defined as *“the long-term abstinence from criminal behaviour among those for whom offending had become a pattern of behaviour”* (McNeill et al., 2012, p. 3). It is evident from these definitions that the models of recovery and desistance share similarities as they are driven by dynamic processes of change rather than passivity (Best et al., 2016; Van Roeyen et al., 2017). Recovery is typically conceptualized in three main stages, early recovery (first year), sustained recovery (1–5 years), and stable recovery (beyond 5 years) (The Betty Ford Institute Consensus Panel, 2007). Similarly, desistance has been characterized by three stages, primary desistance (stopping offending behavior), secondary desistance (involving changes in social networks and social identity), and tertiary desistance (involving recognition and acceptance by a range of societal groups that change has happened) (Maruna & Farrall, 2004; Weaver, 2019). Furthermore, recovery and desistance definitions suggest that the goal in both is to move beyond simple remission of substance use and offending behaviors toward a state of flourishing marked achievement and social contribution (Eddie et al., 2021; Gutierrez et al., 2022; Makin et al., 2022).

### Recovery Capital as the Basis for Understanding the Addiction Recovery Process

The specific meaning of recovery is unique to the recovering individual; however, as the amount of research on addiction recovery has increased, researchers have established key recovery domains, including improved physical and mental health and wellbeing, societal participation and citizenship, abstinence, sobriety or controlled substance use, productive and meaningful living leading to stable housing and income that includes enhancing individuals’ rights and responsibilities (Ashford et al., 2019; Neale et al., 2014, 2015, 2016). The recovery capital model has been defined as the sum of strengths and resources one can use to address alcohol and drug addiction (Granfield & Cloud, 1999). Central to the recovery capital model is the notion that while recovery is about gaining control over problematic substance use, it is also a holistic process of change. Therefore, it is about building resources (capital) across several domains—personal, social, and community (Best & Hennessy, 2022).

### Living in Recovery Housing as a Pathway to Recovery and Desistance

One fundamental assumption of the recovery capital model is that basic needs in the personal recovery capital domain likely need to be met in order to ensure flourishing. As a result, access to stable housing is a core component in initiating and maintaining recovery and desistance (Henley, 2014; Polcin, 2014). Among recovering populations, recovery housing services are usually provided after in-patient or residential treatment in the early stages of recovery as a part of continuing care to ensure that basic recovery needs are met, reducing distress, and assisting the individual in becoming independent in daily life (Reif et al., 2014). Studies conducted among recovering populations have shown that living in a recovery house is generally associated with improved recovery outcomes, such as reduced substance use (Groh et al., 2009; Jason et al., 2007; Mahoney et al., 2023; Polcin et al., 2010; Tuten et al., 2012), reduced psychiatric symptoms (Mahoney et al., 2023), and reduced use of dysfunctional coping strategies (Mericle et al., 2019).

Moreover, as recovery houses typically have groups of residents whose interaction may lead to an increase in social recovery capital, time in recovery houses has been associated with increased quality of life, social support, and improved recovery capital scores (Best et al., 2020, 2023; Cano et al., 2017; Härd et al., 2022). Similar findings have resulted from studies conducted among CLS-involved recovering individuals (Polcin, 2018). These studies have shown that time in a recovery house is associated with a lower rate of post-release recidivism (Hiller et al., 1999), decreases in re-incarceration (Prendergast et al., 2004) and reduced re-arrest, sustained at 18-year follow-up (Martin et al., 2011). Also, living in recovery housing is associated with reduced substance use (Jason et al., 2016), and improved abstinence (Jason et al., 2016) and employment status (Jason et al., 2015, 2016) in this cohort of recovering individuals. Altogether, these findings indicate that recovery houses provide a crucial setting for improving recovery capital and reducing recovery barriers for those in recovery with CLS involvement.

Since living in a recovery house is associated with positive recovery outcomes, retention within recovery housing settings is essential, as premature discharge could lead to less optimal recovery outcomes. The importance of retention in recovery housing as a prognostic to improvements in recovery outcomes has been demonstrated in previous studies (Cano et al., 2017; Härd et al., 2022; Hser et al., 2004; Jason et al., 2007), including for those with CLS involvement (Jason et al., 2015, 2016). However, individual characteristics are associated with retention in recovery housing. In one study, longer retention was associated with being male, older, and having CLS exposure (Hser et al., 2004). Furthermore, a recent study found that greater retention in recovery housing was associated with various factors, such as being male, not being Black or African American, not reporting recent substance use, having fewer unmet needs around housing support, and reporting greater levels of citizenship and community involvement (Best et al., 2023). Overall, several individual-level factors are associated with retention, but more research is needed on how CLS involvement is associated with retention trajectories in recovery homes.

## Study Aims

Residency in recovery housing is associated with positive outcomes among recovering people with and without recent CLS exposure, so understanding retention trajectories in recovery housing is essential. CLS-involved recovery populations may have more recovery barriers and unmet needs and less recovery capital than non-CLS recovery populations (Best et al., 2016; Henley, 2014; Neale et al., 2016; Van Roeyen et al., 2017), yet, the evidence is inconclusive as to whether retention in recovery houses and discharge reasons differ between CLS- and non-CLS-involved recovering populations. Therefore, we aimed to compare retention trajectories and discharge outcomes between criminally and non-criminally justice-exposed individuals residing in recovery housing. Two specific research questions were assessed:

RQ1: What are the time-to-event retention outcomes of a CLS-exposed population residing in recovery housing?
RQ2: How do recovery housing retention outcomes among CLS-exposed residents compare with non-CLS residents?

## Methods

### Participants and Setting—Recovery Houses

We included data from 1,978 recovery house (RH) residents, of which 660 had one intake and one discharge status, and 1,378 had multiple intakes and discharge dates and outcomes (suggesting they entered and exited a recovery residence more than once during the time period of data capture). All participants who had consented to their data being used for research and lived in an RH (n = 101 houses with approximately a total of 1,000–1,150 residents at any given time) certified by the Virginia Association of Recovery Residences (VARR) were included in the study. These recovery residences primarily consisted of single-sex residences. They are located throughout Virginia, primarily in residential neighborhoods, with a mean of 9.4 residents (range of 3–21). VARR residences operate at Level II, that is democratically run by residents with a recovering peer as an on-site manager and Level III, that also provides on-site support services and has paid staff (Polcin, 2018). All VARR residences are certified by the National Alliance for Recovery Residences (2018). There are two means by which a person with a CLS history may access certified recovery housing in Virginia. The first is direct from incarceration (e.g., pre-adjudication before a final court decision), and the second is for those with an offending history who have not been directly referred from the CLS. For the second group, the placement results from a joint decision between the individual, the VARR team and the RH given factors such as gender, age, and location.

### Instrument

The REC-CAP questionnaire (Best et al., 2023; Cano et al., 2017) measures recovery capital, recovery barriers, and unmet needs. The questionnaire was found to have acceptable psychometrics (Cano et al., 2017) and is certified by a National Alliance of Recovery Residences (NARR) affiliate, the Florida Association of Recovery Residences (Best et al., 2023). The REC-CAP incorporates previously validated questionnaires: Assessment of Recovery Capital (ARC, Groshkova et al., 2013b) encompassing personal and social recovery capital; Recovery Group Participation Scale (RGPS, Groshkova et al., 2013a) to assess community recovery capital; Commitment to Sobriety Scale (CSS, Kelly & Greene, 2014); and the Wellbeing measures and Barriers to Treatment Scale from the Treatment Outcomes Profile (Delgadillo et al., 2013).

### Predictors: CLS Versus Non-CLS Groupings

The analysis aimed to understand retention and discharge for CLS clients within recovery housing compared to non-CLS clients. We defined a CLS client as any individual who reported exposure to the criminal legal system in their baseline completion of the REC-CAP in the last 90 days. This was determined through an individual’s REC-CAP record where the respondent had answered “yes” to any of the following in the previous 90 days: (a) any involvement with the police (*n* = 400, *n* = 20.2%); (b) on probation (*n* = 960, 48.5%); (c) on parole (*n* = 51, 2.6%); and (d) any other CLS involvement (*n* = 346, 17.5%). We relied on the phrasing within REC-CAP that focused on “any involvement” rather than a specific question on arrests, which aimed to avoid stigmatizing respondents and thus could be interpreted in multiple ways.

Given that a large proportion of recovery housing residents have broader exposure to the CLS, we assume that a positive response to this question is likely to focus on issues relating to previous or current offending (also given the close relationship between this question and admission in recent offending which is shown below). However, further work is required to test this supposition. Within this grouping, recent involvement in offending was just under one-fifth (*n* = 377, 19.1%), of which only 13 individuals reported involvement in offending but with no current involvement with the CLS. Overall, 60.3% (*n* = 1,192) of the total population had recent exposure to at least one part of the CLS in the last 90 days.

### Outcomes

The discharge outcomes of this study were categorized into three categories. The first discharge outcome is a “successful completion” as agreed by the resident and the residence manager, encompassing the retention and completion of drug and/or alcohol support programs within the RH. The second outcome is a “premature discharge” or “negative discharge,” including instances when an individual abandoned the program voluntarily, had a criminal justice discharge (leading to further CLS involvement), died, or other involuntary discharges and instances of relapse leading to eviction from the RH. The third outcome is a “neutral discharge,” including individuals who had changed network/partner, had a medical discharge or were referred out of the RH to another agency.

We fitted Competing-Risk regression models correlating the three discharge outcomes to 14 predictor variables plus a client group defined as “non-CLS” or “CLS.” We deployed a quasi-experimental approach to understand outcomes between CLS-involved and non-CLS-involved samples. Thus, the results are interpreted in terms of marginal effects, using a counterfactual framework to compare participants with three discharge outcomes (i.e., if all participants were CLS clients vs. if all participants were non-CLS clients). To ease interpretation, we describe “CLS clients” and “non-CLS clients” to describe this counterfactual framework, such that everyone would be in one group or another. We aggregated multiple records per client into a single record by computing the total number of days in residency summing over multiple discharge episodes. As for the value of the predictors and discharge outcome, we attributed the last recorded value to an individual’s record, for example, the outcome closest to the discharge date.

## Analytical Strategy

### Competing Risks Analyses

Time-to-event studies examine the effect of an intervention on the time taken for an event to occur. Kaplan–Meier curves are appropriate for only one event of interest. However, when multiple different outcomes are of interest, a competing risk (CR) issue occurs when events are mutually exclusive, that is, where one event precludes any other event from occurring. In a CR setting, the cumulative incidence function (CIF) calculates the probability of experiencing a specific event before a particular time and before a competing event occurs. This implies that a subject undergoing a competing event is determined to be not at risk of experiencing alternative outcomes (Schuster et al., 2020). To support these survival analyses, the restricted mean survival time (RMST) metric further estimates the average event-free time from a time origin to a pre-specified follow-up length (Royston & Parmar, 2011).

Studies deploying CR in CLS settings have examined parole violations (Grattet & Lin, 2016; Ostermann et al., 2020), probation supervision models (Lattimore et al., 2016), the relationship between CLS supervision and overdose mortality (Binswanger et al., 2020), recidivism including arrest (Bolger, 2018), and reconvictions for sexual offences (Escarela et al., 2000). Thus, for this study, the use of CR methodology is appropriate to understand outcomes from clients residing in recovery housing where effects may include a successful outcome, such as completing a treatment program, or “failures,” such as dropping out, involuntary discharges, death in residency, and referral to other support services.

### Cause-Specific Cumulative Incidence Function

We focus on the Cumulative Incidence Function (CIF), which accounts for the fact it is impossible to experience an event if a competing event is experienced first (Collett, 2023); for example, a premature discharge from an RH will preclude a client from completing treatment. This study has three competing outcomes: successfully completed treatment, neutral reasons for discharge, and premature/negative discharge. Clients still in the RH are considered right-censored records (Cleves et al., 2016). We use a dedicated command –stpm2- to account for CR in the Stata software (version 16) to fit the models (Collett, 2023). The modeling tool fits flexible parametric survival models without the constraint of proportional hazards (Royston & Lambert 2011). As recommended by Mozumder et al. (2021), we fitted models on the log-cumulative hazard scale with five degrees of freedom (df) for the baseline cause-specific splines. All predictors were fitted as Time-Varying Covariates (TVC), including the client group term, so its effect changes as follow-up time progresses (Collett, 2023). Thus, hazard rates are no longer in a constant ratio over time, so a single hazard ratio cannot be used as an effect measure (Hernán, 2010).

By adopting a quasi-experimental approach (Mozumder et al., 2021), we fit a competing risks model to each of the three competing events, followed by the marginalization of effects using the command -standsurv- (Lambert 2021; Syriopoulou et al., 2022). The interpretation of results is in terms of marginal effects, using a counterfactual framework to compare three discharge outcomes had everyone in this study been CLS clients versus had everyone in this study been non-CLS clients. Rather than creating the counterfactual group using a matching process, the –standsurv- command forms model-based predictions for all individuals in the study twice, once with all individuals assigned to the non-CLS group and repeated for every individual then assigned to the CLS group. By using this approach, predictor distributions across the groups compared are balanced to approximately equivalent groups on key confounding variables because the predictive distributions are forced to be identical between counterfactual groups (Lambert, 2021).

### Restricted Mean Survival Time (RMST) and Restricted Mean Time Lost (RMTL)

The restricted mean survival time (RMST) estimates the average event-free time until a pre-specified follow-up length (Royston & Parmar 2011). The RMST quantifies the expected length of residency in RH during the first 30 months of follow-up, a cut-off time chosen as the longest stay in RH over an extended period (e.g., over multiple events). The restricted mean time lost (RMTL) complements the RMST and quantifies the expected length of time not spent in residency during the first 30 months of follow-up. The RMTL is applied to a composite event in the presence of competing risks to apportion its components to each discharge outcome (Andersen 2013). The individual components are called cause-specific RMTL (Conner & Trinquart 2021). The effect measures for this metric are the estimated difference between the two marginal RMTLs and the estimated difference between each marginal cause-specific RMTL, presented with 95% confidence intervals (Gardner & Altman 1986).

## Findings

Table 1 provides summary statistics on baseline characteristics grouped by three types of discharge outcomes and includes clients who remained in the RH at the final assessment.

**Table 1.** Summary Statistics by Discharge Outcome.

	Retained in RH (*n* = 621)	Completed (*n* = 174)	Neutral (*n* = 510)	Negative/ Premature (*n* = 673)
	*M* (*SD*)	*M* (*SD*)	*M* (*SD*)	*M* (*SD*)
Age	38.0 (12.0)	38.6 (10.9)	36.6 (10.7)	36.1 (10.6)
Psychological health (0–20)	13.7 (4.7)	13.7 (4.7)	12.7 (5.1)	13.4 (4.5)
Physical health (0–20)	14.3 (4.6)	14.6 (4.3)	13.8 (4.8)	14.3 (4.5)
Quality of life (0–20)	13.0 (5.1)	13.7 (4.8)	12.0 (5.1)	12.4 (4.9)
Accommodation (0–20)	14.4 (5.5)	14.9 (5.4)	14.0 (5.4)	14.4 (5.4)
Client social support (0–28)	21.8 (6.3)	22.6 (5.8)	20.2 (6.5)	21.0 (6.6)
Commitment to sobriety (0–30)	28.9 (2.9)	28.6 (3.0)	28.3 (4.0)	28.4 (3.8)
Unmet needs (0–8)	2.5 (2.2)	2.1 (2.2)	2.4 (2.1)	2.5 (2.0)
Barriers (0–5)	1.9 (1.1)	1.9 (1.0)	2.1 (1.1)	2.2 (1.1)
RGPS (0–14)	7.4 (5.1)	7.8 (4.7)	7.5 (5.0)	6.7 (5.0)
ARC (0–50)	38.0 (10.4)	39.1 (9.8)	36.4 (10.2)	37.3 (10.5)
	Number (%)	Number (%)	Number (%)	Number (%)
Gender				
Female	222 (35.7%)	58 (33.3%)	216 (42.4%)	214 (31.8%)
Male	399 (64.3%)	116 (66.7%)	294 (57.6%)	459 (68.2%)
Ethnicity				
Non-Caucasian	208 (33.5%)	52 (29.9%)	145 (28.4%)	257 (38.2%)
Caucasian	413 (66.5%)	122 (70.1%)	365 (71.6%)	416 (61.8%)
Group				
Non-CLS	229 (36.9%)	57 (32.8%)	219 (42.9%)	211 (31.4%)
CLS	392 (63.1%)	117 (67.2%)	291 (57.1%)	462 (68.6%)

Summaries of RH residents by discharge outcome show that the “neutral” and “premature/negative” groups have a broadly similar profile and tend to present with lower recovery capital (lower psychological health, quality of life, support networks, being younger and reporting more barriers) than the “completed/successful” and “retained in RH” groups. Individuals with a “neutral” discharge were likelier to be female and Caucasian, reporting lower baseline ARC scores and no CLS exposure in the previous 90 days.

Table 2 shows crude differences in the mean length of stay in an RH by outcome, where shorter residency averages can be noted for CLS clients comparing individuals retained in RH (182 days compared to 202 for non-CLS clients) and those who completed their residency (212 days compared to 236 for non-CLS clients). CLS clients reported longer mean stays if they had a neutral outcome (106 days compared to 89 for non-CLS clients) and a premature/negative outcome (85 days compared to 71 for non-CLS clients). Summary means for the CLS and non-CLS groups were similar: 131 days compared to 132 days, whereas summary medians differed by two weeks: 79 days compared to 93 days.

**Table 2.** Summary Mean and Median Residency Length (Days) by Client Group.

	Non-CLS	CLS
	Days (*SD*)	Days (*SD*)
Retained in RH	202 (163)	182 (146)
Completed	236 (177)	212 (137)
Neutral	89 (122)	106 (137)
Premature/negative	71 (76)	85 (93)
Total mean residency length (days)	131	132
Total median residency length (days)	79	93

### Cause-Specific Differences Between Cumulative Incidence Functions by Client Group

**Figure 1.**
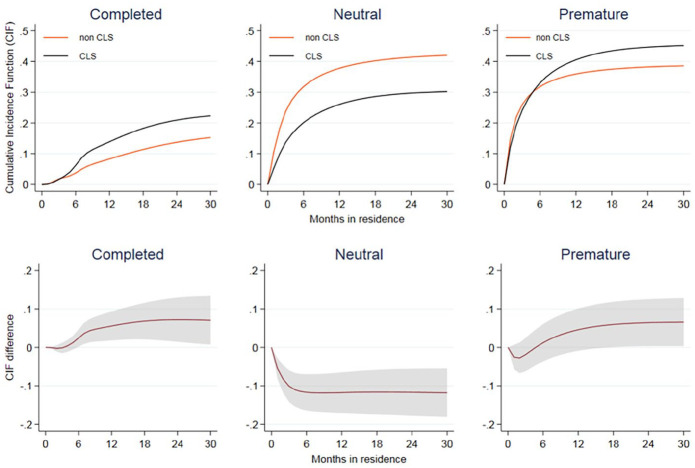
Estimated marginal cause-specific CIF (top row) and marginal CIF differences: CLS versus non-CLS clients by discharge outcome, with 95% CI (bottom row).

At 30 months follow-up, CLS clients were more likely to have achieved a completed/successful or premature/negative discharge but not a neutral discharge than non-CLS clients. Specifically, the estimated marginalized CIF indicates that if everyone were a CLS client (top left panel, Figure 1), 22.4% (95% confidence interval [CI] 18.4, 26.9) would achieve a completed discharge. The estimated difference in standardized cause-specific CIF between groups shows that, compared to everyone in this study being a non-CLS client, if everyone were a CLS client, there would be an increase of 7.1 percentage points (95% CI 0.7, 13.4) in successfully completed discharges (bottom left panel, Figure 1). Yet, this risk difference for completed/successful discharges only appears after 6 months. It increases steeply until 12 months and shows a slowing increase until 18 months, after which it seems to stabilize.

### Marginal Cause-specific Restricted Mean Time Lost by client group

**Figure 2.**
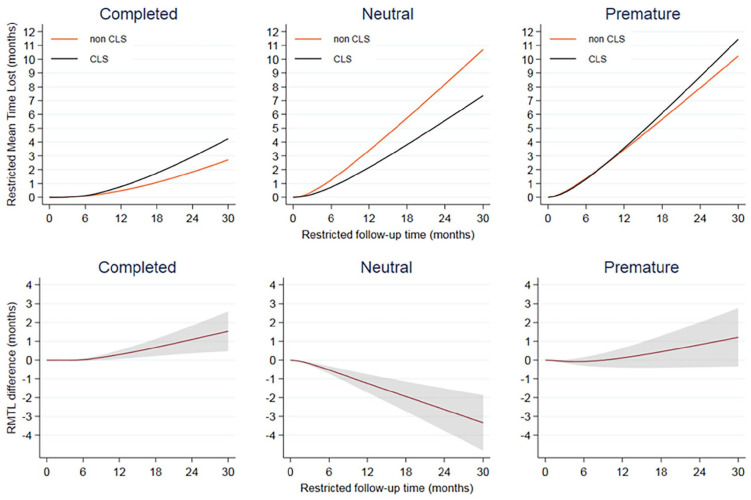
Estimated marginal cause-specific RMTL (top row) and marginal RMTL differences: CLS versus non-CLS clients by discharge outcome, with 95% CI (bottom row).

At 30 months follow-up, the estimated marginal RTML show that if everyone in the study were a non-CLS client (top left panel, Figure 2), the RMTL due to a completed/successful discharge would be 2.7 months (95% CI 2.1, 3.5); if everyone were a CLS client, the RMTL due to a completed/successful discharge would be 4.2 months (95% CI 3.6, 5). Second, if everyone in the study were a non-CLS client (top center panel, Figure 2), the RMTL due to a neutral discharge would be 10.7 months (95% CI 9.6, 12.0); if everyone were a CLS client, the RMTL due to a neutral discharge would be 7.4 months (95% CI 6.6, 8.2). Finally, if everyone in the study were a non-CLS client, the RMTL due to a premature/negative discharge (top right panel, Figure 2) would be 10.2 months (95% CI 9.1, 11,5); if everyone had been a CLS client, the RMTL due to a premature/negative discharge would be 11.4 months (95% CI 10.6, 12.3). Consequently, non-CLS clients’ total RMTL is 23.6 months (95% CI 22.9, 24.4); hence their RMST (discharge-free) is 6.4 months (95% CI 5.6, 7.1). Yet, in comparison, CLS clients’ total RMTL is 23 months (95% CI 22.5, 23.6), resulting in the RMST (discharge-free) being 7 months (95% CI 6.4, 7.5). Thus, the difference in marginal RMTL between CLS and non-CLS clients is estimated to be only 0.6 months (95% CI 1.6, 0.4).

### Differences Between Cause-Specific RMTL

At 30 months follow-up, the estimated difference in marginal cause-specific RMST between groups shows that, compared to everyone in this study being a non-CLS client, if everyone were a CLS client, there would be (a) an increase of 1.5 months in RMTL due to a completed/successful discharge (bottom left panel, Figure 2); (b) a decrease of 3.3 months in RMTL due to a neutral discharge (bottom centre panel, Figure 2]; and (c) an increase of 1.2 months in RMTL due to a premature/negative discharge (bottom right panel, Figure 2).

## Discussion

Our primary aim was to assess whether individuals entering recovery residences with recent CLS involvement had differential retention patterns and whether there were systematic differences in the length of stay and reasons for discharge. Our findings using competing risks analyses present a nuanced set of conclusions. While the overall mean length of stay was broadly similar (at 131–132 days), which is in itself an encouraging finding, those who entered recovery residences with recent justice involvement were more likely to have both a completed/successful (positive) and a premature (negative) discharge but were less likely to have “neutral” reasons for release. The findings suggest retention is linked to mandated requirements associated with criminal justice conditions. This finding indicates that if CLS clients can be encouraged to stay and comply with recovery residence rules, they may be more likely to complete the program and be less likely to move to other residences or have other “neutral” outcomes. This may also reflect the lack of alternative, safe accommodation for CLS-exposed residents nearing discharge (DeGuzman et al., 2019), making retention the only viable choice. Furthermore, fewer “neutral” outcomes among the CLS-exposed group may also reflect a lack of prosocial ties, where non-CLS-exposed individuals may be able to transfer to other recovery houses and services available through family and other connections. Given the evidenced benefits for retention in recovery residences for criminal justice outcomes (Hiller et al., 1999; Prendergast et al., 2004) and the scale of drop-out in recovery residences, this is a crucial issue for research, and the current findings imply that retention patterns and discharge reasons are linked to mandated requirements associated with criminal justice conditions.

The other key finding concerns time to discharge, with CLS clients less likely to discharge themselves prematurely in the first 6 months but thereafter having a higher risk of premature/negative discharge with a sharp increase up to 18 months. This finding may be associated with their probation conditions in this period (e.g., requiring an individual to complete a supervised treatment program and possibly be linked to specific supervisory requirements of probation orders). This decrease in the intensity of supervision over time may also affect the availability of secondary social support, leading to more negative outcomes. It may also be the case that non-CLS clients have greater freedom to “shop around” and transfer to other recovery residences. However, CLS clients lacking this opportunity could be more likely to express dissatisfaction through unplanned discharge. There may be a sense that the stakes are higher, and so CLS clients may be more likely to endure aspects of recovery residence life that they do not like to fulfil their justice requirements, but this “double-edged sword” may also lead to an increased sense of pressure and stress that may cause them to leave.

Contrary to our expectations is the finding that CLS clients are more likely to have a completed/successful discharge than non-CLS clients. This is a crucial and new finding as better retention, and positive outcomes are associated with higher recovery capital (consistent with Härd et al., 2022). This aligns with other research suggesting that navigating the initial 6 months is pivotal for CLS clients (Wooditch et al., 2014). This may involve more intensive support (Belanger et al., 2024), in that the initial 6-month window provides a critical period for effective justice disposals to recovery residences and to prepare individuals to establish personal and social recovery capital that address criminogenic needs (Best et al., 2024). Continuity of care and congruence of provision between justice and community settings has been established as critical for the effectiveness of Therapeutic Communities (Hiller et al., 1999; Vanderplasschen et al., 2014). It may be similarly crucial for recovery residences.

The importance of the duration of stay is not limited to this first six-month window. We show that 6–18 months from baseline assessment for CLS clients is a period of a heightened risk of a premature/negative discharge from an RH (including discharge for disciplinary reasons). Further mixed methods research is needed to establish the critical factors around justice influences, including both direct, in the form of probation conditions and conditional sentencing, and indirect, around the pathways and experiences of individuals with the added burden and pressure of ongoing involvement in the justice system. There is an added premium to society (Best et al., 2016) where recovery is also accompanied by criminal desistance in terms of reduced costs of justice processing, reduced harm to society and reduced damage to families and communities.

There are several limitations to the current study. The analysis is of person-level administrative data and does not include information about the residents’ motivations, experiences of residence living or departure, or post-departure experiences (including reoffending), and future studies should seek to include these to augment the analyses. Also, there is no information on aspects of the residences that could strongly influence client retention (e.g., social climate, staffing characteristics, staff-client dynamics, neighborhood environment and safety, or the characteristics and roles of peer recovery specialists in Level 2 and Level 3 recovery houses). We have also aggregated self-reported categories of criminal justice involvement (probation, involvement with police, other justice system involvement, and self-reported offending). Therefore, we did not examine individual-level pressures on returning to court or prison that may have informed decisions to stay or leave the residence, or information on the specific mandates that may prevent or discourage early departure from recovery residences. For example, further work is required to determine the level of CLS supervision provided to each criminal justice-exposed client. The paper aimed to assess the overall effect of CLS exposure on retention in a recovery house. There may be sub-groups of offenders (e.g., those on parole, etc.) that may benefit from more granular analysis. Furthermore, it is important to note that our categorization of “involvement with the police” was broad, including all potential contact with police. Future research may use a measure of police involvement that captures the seriousness of the encounter (e.g., speeding ticket vs. robbery). Finally, our categorization of “positive” (i.e., completed/successful), “neutral,” and “premature”/“negative” is inevitably both judgment-laden and externally imposed, and future research should look to refine these terms.

Nonetheless, this paper addresses the vital issue of retention and completion rates in recovery residences for a vulnerable and marginalized population. While we know that recovery residences have demonstrated effectiveness (Härd et al., 2022; Jason et al., 2007, 2016; Reif et al., 2014), the effectiveness appears to be strongly linked to adequate “doses” of the intervention (Jason et al., 2016). A greater understanding of the nuances of reasons for leaving and the timing of departures is essential, which has largely been neglected in the study of Level 2 and Level 3 NARR-affiliated residences. This paper moves the field further by using competing risks in time-to-event models to establish a statistical proxy for a randomized controlled trial (RCT) in an area where an RCT would not be possible and to demonstrate that there are highly nuanced and systematic differences between justice and non-justice residents in the timing and reasons for their departures from recovery residences.
